# The Spectrum of Genetic Mutations Among Patients with Hereditary Breast and Ovarian Cancer

**DOI:** 10.3390/jcm14134536

**Published:** 2025-06-26

**Authors:** Amani Al Hajeri, Amna Al Awadhi, Nitya Kumar, Ghufran Jassim

**Affiliations:** 1 Genetics Center, Government Hospitals, Manama P.O. Box 12, Bahrain; aawadhi4@health.gov.bh; 2 The Royal College of Surgeons in Ireland, Medical University of Bahrain, Busaiteen P.O. Box 15503, Bahrain; nkumar@rcsi-mub.com (N.K.); gjassim@rcsi-mub.com (G.J.)

**Keywords:** breast cancer, familial cancer, genetics, hereditary breast cancer, ovarian cancer

## Abstract

**Background:** Breast cancer (BC) is the leading female cancer globally, with Bahrain having the highest incidence in the GCC. In this study, we aimed to identify and describe the high-risk pathogenic/likely pathogenic mutations in a cohort of 160 Bahraini patients who underwent genetic testing for hereditary cancer susceptibility genes. **Methods**: This study included 160 women referred to Bahrain’s Government Hospitals for genetic analysis between January 2021 and May 2024. All women underwent NGS cancer gene panel testing. Demographic and clinical data were recorded for each patient. Categorical variables were described using frequencies and percentages, and continuous data was depicted using means and standard deviations or medians. **Results**: Pathogenicity was significantly higher in individuals with high-risk mutations compared to those with medium- and low-risk mutations. However, mortality was highest among those with medium-risk mutations, exceeding that of both the high- and low-risk groups. **Conclusions**: In Bahrain, the genetic profile of BC germline mutations aligns closely with international data. However, further research is needed to assess moderate- and low-risk mutations and their pathogenicity within the diverse ethnic populations of the Middle East.

## 1. Introduction

Breast cancer (BC) is the most frequently diagnosed malignancy and the leading cause of cancer deaths in females worldwide, with an estimated 2.3 million new cancer cases (one in four new cancer cases) and 685,000 cancer deaths (one in six deaths) in 2020 [1]. It is a multifactorial disease caused by a complex interplay between genetic and environmental/lifestyle factors [2]. It is currently estimated that hereditary breast and ovarian cancer (HBOC) accounts for 5–10% of all BC cases and is inherited in an autosomal dominant manner. The large number of cases of HBOC syndrome is caused by the presence of germline mutations in either the BRCA1 or the BRCA2 gene located on chromosome 17q21 and 13q12-13, respectively [3]. These two high-penetrant genes, which act as tumor and cell growth suppressors, are fundamental for normal cell function and genomic stability.

Compared to other Gulf Cooperation Council (GCC) states, Bahrain has the highest incidence of BC, which accounts for 44.4% of all female cancers in 2022 [4]. The estimated incidence of BC in Bahrain is 117 per 100,000 women, and the age-standardized incidence rate (ASR) per 100,000 is 58.5, exceeding that for all other GCC countries [4,5]. For instance, in Saudi Arabia, the ASR is three times less than in Bahrain (ASR 19.76) [6]. As BC can be attributed to many risk factors including atmospheric pollution [7], it is difficult to pinpoint the cause of the remarkable increase in Bahrain. Albeshan S. et al. stated that the overall mean age at diagnosis of BC in GCC countries was 50.6 years compared to 60 years in Western countries [8]. In Bahrain, the overall mean age at diagnosis ranged between 50.9 and 51.8 years [5,9], respectively.

*BRCA1-* and *BRCA2-*associated hereditary breast and ovarian cancer (HBOC) is characterized by an increased risk for male and female BC, ovarian cancer (including fallopian tube and primary peritoneal cancers), and, to a lesser extent, other cancers, such as prostate, pancreatic, and melanoma [10]. However, not all cases of HBOC can be attributed to *BRCA1* and *BRCA2*, as more than 20 other genes have been associated with an increased risk of familial breast and/or ovarian cancer [11].

In previous years, BC genetic testing for women with family histories of breast or ovarian cancer has become a part of clinical practice in many countries. However, the test is generally limited to *BRCA1/2* mutations, and no further investigation is offered for patients with negative results. Recently, exome sequencing has uncovered substantial locus heterogeneity among affected families without BRCA1 or BRCA2 mutations [11]. Numerous studies have shown that germline pathogenic variants in genes like ATM, BARD1, BLM, CDH1, CHEK2, MUTYH, PALB2, PTEN, RAD51C, RAD51D, TP53, and others are associated with an increased BC risk [12]. One of the most recognized testing policies is the National Comprehensive Cancer Network (NCCN) guidelines, which recommend using risk stratification when selecting both unaffected and affected women for testing [13].

Establishing the presence of pathogenic mutations in BC patients is important, both in terms of imaging diagnosis and screening for the affected as well as the non-affected family member and in the therapeutic management and subsequent follow-up of these patients [14].

Studies on the prevalence of these high-penetrance mutations in the Bahraini population are limited and are mainly focused on pathogenic or potentially pathogenic mutations in the BRCA1 and BRCA2 genes [15]. A recent study investigated the germline variants in 54 Bahraini women with BC using a next-generation-sequencing-based multigene panel, which yielded additional genomic information on low-penetrance genes, although the clinical significance of these genes has not been fully appreciated yet [16].

Our study aimed to identify and describe the high-risk pathogenic/likely pathogenic mutations in a cohort of 160 Bahraini patients who were referred for genetic testing either because they fit the NCCN criteria for genetic testing or they have a high risk of having HBOC.

In Bahrain and other GCC countries that share similar demographic, cultural, and lifestyle traits as well as healthcare systems, these results will serve as a cornerstone for hereditary BC risk-assessment programs. They will also provide a framework for screening, genetic testing, and the management of familial BC.

## 2. Materials and Methods

This study used a retrospective cohort of women referred to the Genetics Center, Government Hospitals (Bahrain) between 1 January 2021 and 15 May 2024. The data was accessed starting from 6 March 2024 to 30 September 2024. The sample included 160 women who were referred to and consulted at the Genetic Center/(GH) in Bahrain. All included women had their blood tested with an NGS cancer gene panel, regardless of their family history of breast or any other malignant tumors. The clinical diagnosis and classification of BC were in accordance with the World Health Organization criteria. These patients had undergone genetic testing as per the Genetic Center and Pathology Department’s policy, which is in line with the NCCN guidelines. The results were recorded at the genetic center database. All patients provided informed written consent to have genetic data from their medical records used in research.

Demographic and clinical data were recorded for each patient. This study was conducted in accordance with the Helsinki declaration. Ethical approval was obtained from the Research and Ethics Committee of GH and the Research and Ethics Committee of the Royal college of Surgeons in Ireland-Bahrain.

### 2.1. Inclusion Criteria

This study included women referred to the Genetic Center at the GH for genetic analysis because they are Bahraini women between the ages of 18 and 74 years and fitting one or more of the following criteria: early onset BC (less than 50 years), triple negative BC, bilateral or recurrent BC, breast and other primary cancer, personal history of ovarian cancer, at-risk, cancer-free patients with a family history of malignancy (according to the NCCN guidelines), at least one first-degree relative or two second-degree relatives with BC, metastatic BC for therapeutic decisions.

### 2.2. Exclusion Criteria

Patients were excluded if they were non-Bahraini, male (because there were none during the data collection timeframe), or if genetic testing was performed for reasons not aligned with the NCCN guidelines.

In this study, we used NGS panels of 70–143 susceptibility BC genes provided by overseas partners through the GH (Centocancer^®^ panel-Am Strande 7, 18055 Rostock, Mecklenburg-Vorpommern Germany/Medgenome comprehensive cancer panel^®^-Bangalore South, Karnataka 560100, India). These genes were selected based on the available evidence of their association with hereditary cancer syndromes [17,18].

We classified our patients’ pathology findings according to the World Health Organization (WHO) classification of breast tumors [19].

### 2.3. Genetic Testing Workflow

Genomic DNA was isolated from EDTA-anticoagulated whole blood (Qiagen QIAamp DNA Mini-Qiagen Straße 1, 40724 Hilden, Nordrhein-Westfalen, Germany). Library preparation used hybrid-capture commercial kits (Centogene CentoCancer^®^ and MedGenome Comprehensive Cancer Panel^®^). Target enrichment covered 70–143 cancer-susceptibility genes with ≥99% of bases at ≥100×. Sequencing was performed on Illumina NextSeq 550 instruments (5200 Illumina Way, San Diego, CA 92122, USA). Reads were aligned to GRCh38 with BWA-MEM; variant calling employed GATK v4.2 HaplotypeCaller; annotation utilized Illumina VariantInterpreter v2.17.0, ClinVar 2024-01, gnomAD v4, and in-house Middle-East frequencies. Variants were classified using the ACMG/AMP 2015 criteria. Orthogonal Sanger confirmation was performed for all (likely) pathogenic findings.

### 2.4. Statistical Analysis

We described continuous data as either the mean ± SD or median [IQR] where appropriate. Categorical variables were described using frequencies and percentages and analyzed using either Pearson χ^2^ or Fisher’s exact tests. Normality for continuous variables was assessed using the Shapiro–Wilk test. Analysis for continuous variables was performed using either a Student’s t or Mann–Whitney U test where appropriate. Survival was estimated with the Kaplan–Meier method. Hazard ratios and corresponding 95% confidence intervals were estimated using multivariable Cox proportional-hazards models adjusted for age at diagnosis and family-history burden. Analyses were executed in SPSS v26 and Stata 18; *p* < 0.05 was considered significant.

## 3. Results

The characteristics of the study participants are summarized in Table 1. Among the 160 patients included, 26 (16%) were at-risk, cancer-free, while 134 (84%) were already diagnosed with cancer. The mean age of all participants was 54.58 years (SD 11.32 years), while the mean age at diagnosis was 39 years (SD 19.15 years), with 63 patients (47%) diagnosed between the ages of 30 and 49 years. Only four patients (3%) were diagnosed before the age of 29 years.

When asked about a family history of cancer, 135 patients (84%) reported a positive family history, while 23 (14%) had none. Among those with a positive family history, 71 (44%) had first-degree relatives diagnosed with one of the following: BC before the age of 50 years, ovarian, pancreatic, prostate, or male BC. Eight participants (5%) reported at least three affected family members on the same side of the family with breast, prostate, ovarian, or pancreatic cancer. Similarly, eight patients (5%) had first- or second-degree relatives with colon or uterine cancer. Additionally, 48 patients (30%) had other blood relatives with cancer.

Of the affected participants, 109 (81%) had only BC, 5 (4%) had only ovarian cancer, and 20 (15%) had a combination of BC and other cancers, including ovarian cancer.

Among patients with BC, 101 (77%) had unilateral breast involvement, while 30 (23%) experienced either recurrence or bilateral breast involvement. The majority of patients, 97 (89%), were not classified as triple-negative, whereas 12 (11%) were classified as triple-negative.

In terms of pathology, 54 BC cases (74%) were diagnosed as invasive ductal carcinoma, 12 (16%) were diagnosed as invasive lobular carcinoma, 2 (3%) were diagnosed as in situ intraductal carcinoma, 1 (1%) was diagnosed as in situ lobular carcinoma, and 1 was diagnosed (1%) as invasive papillary carcinoma.

BRCA1 mutations were identified in ten participants (17%), BRCA2 mutations were identified in eight (14%), and ATM mutations were identified in five (8%). Mutations in MSH2 and CHEK2 were found in three patients each (5%), PALB2 mutations were found in two patients (3%), and TP53 and MSH6 mutations were found in one patient each (2%). Additionally, 26 patients (44%) had mutations in other lower-risk genes.

Of all mutations detected, 23 (39%) were classified as pathogenic, while 36 (61%) were variants of unknown significance (VUS). Among the pathogenic mutations, 18 (32%) were categorized as high risk, 10 (17%) were classified as moderate risk, and 30 (51%) were classified as low risk.

Among those with cancer, 22 patients (16%) had metastases, while 111 (83%) did not. At the time of the study, 151 participants (94%) were alive, and 9 (6%) had passed away.

**Figure 1:**
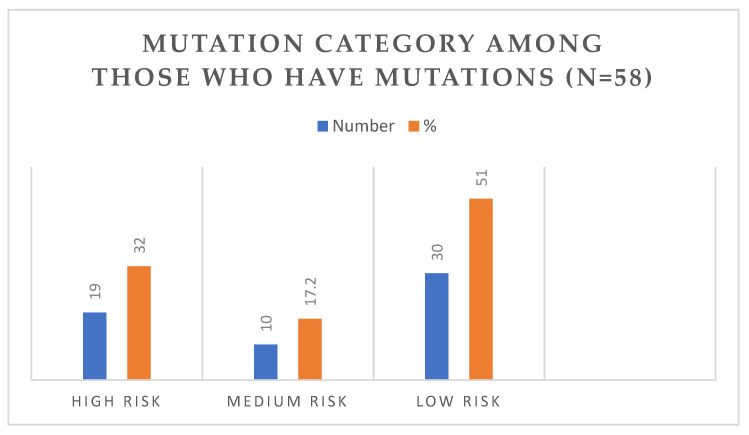
Mutation category among those who have mutations (N = 58).

With respect to the categorization of risk of the individual mutations (Figure 1), it was found that 19 participants (32%) had mutations in the high-risk category, 10 participants (17.2%) had medium-risk mutations, and 30 participants (51%) had low-risk mutations.

A comparison of sociodemographic and clinical characteristics across those affected by cancer and those who were not revealed that the two groups were comparable, with the exception of family history and age distribution (Table 2). All the at-risk, cancer-free participants (n = 26) had a family history of BC compared to 81.3% of those affected with cancer (*p* = 0.056). In terms of the type of family history, most of the at-risk, cancer-free participants (n = 23, 88.5%) had a first-degree relative with breast, ovarian, pancreatic, or prostate cancer compared to only 35% of those with cancer (*p* < 0.001).

The clinical and sociodemographic attributes were compared across categories of mutations as well (Table 3). Pathogenicity was significantly higher (72%) in those with high-risk mutations versus those with medium- (30%) and low-risk (20%) mutations at *p* < 0.001. Mortality was highest in those with medium-risk mutations (20%) compared to those with high-risk (16.7%) and low-risk (10%) mutations (*p* = 0.004). Similarly, the history of a first-degree relative with cancer was highest in those with medium-risk mutations (70%) compared to high-risk (60%) and low-risk (40%) mutations, with *p* = 0.033. Lastly, more of the medium-risk participants metastasized (40%) compared to the high-risk (22%) and low-risk (30%) participants (*p* = 0.006). Tumor pathology, laterality, and triple-negative subtype were similar across the three categories of mutations.

Although the participants with high-risk mutations (Table 4) were diagnosed much earlier (mean age = 37 years) compared to those with medium- and low-risk mutations (46 years and 43 years, respectively), the difference was not statistically significant (*p* = 0.250). Similarly, those with at least three family members with cancers on the same side of the family were diagnosed at a significantly younger age (mean age at diagnosis = 34.29 years, *p* = 0.017) compared to those with other types of family history of cancer. Those with first-degree relatives were diagnosed at a similarly young age (mean age at diagnosis = 35.88 years).

**Figure 2:**
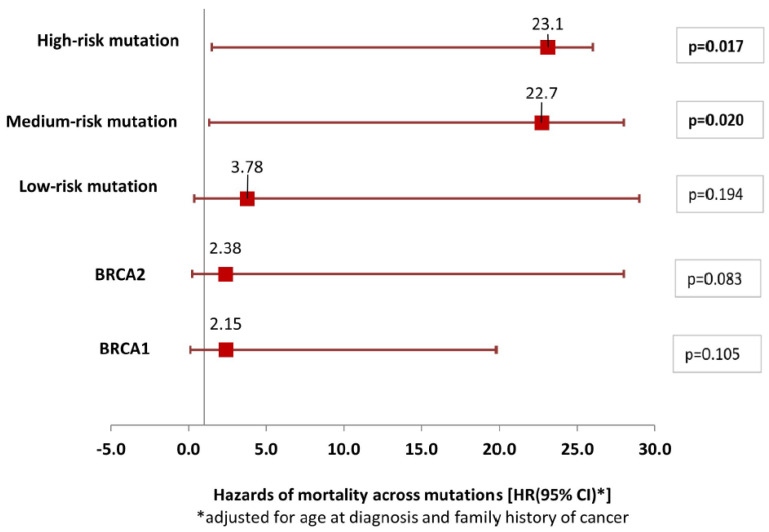
Hazards of mortality across mutation types (N = 128). Hazards of mortality across mutation types (N = 128) after adjusting for age at diagnosis and family history.

Hazard ratios comparing mortality across types of mutations (Figure 2) found that high-risk mutations were significantly associated with mortality (HR = 23.1, =0.017) after adjusting for age at diagnosis and family history. Similarly, medium-risk mutations were also found to have significantly higher hazards of death (HR = 22.7, *p* = 0.020). Given the low numbers of death, the confidence intervals were quite wide, indicating low precision of the estimates.

## 4. Discussion

In this study, we reviewed patients for whom a comprehensive NGS cancer panel was requested, primarily based on the NCCN guidelines. In total, 160 participants fulfilling the criteria were included. The mean age at diagnosis was 39 years, aligning with the observed trend of a younger age at BC diagnosis in Bahrain and the GCC [8]. However, this is notably lower than the average age at diagnosis reported for the general population of women with BC. This discrepancy is likely due to the inclusion of a specific subtype of women in our study cohort.

Our cohort confirms a 39% pathogenic and likely pathogenic P/LP mutation rate, with BRCA1/2 accounting for one-third. Pathogenicity was enriched among high-risk genes, yet mortality was highest in the medium-risk group—echoing emerging evidence that ATM/CHEK2/PALB2 aberrations may portend adverse outcomes unless detected early.

In comparison to studies in other GCC countries, a Saudi (Western Province) cohort found 24.4% of 209 patients carried P/LP variants, with BRCA1 predominance and a strong association with triple-negative disease [20]. In the United Arab Emirates (Dubai cohort, 2017–2022), among 443 mixed-ethnicity patients, 15% harbored BRCA1/2 P/LP, and a further 4% carried actionable non-BRCA variants [21]. High-quality prevalence data remain scarce in Qatar and Kuwait, but a 2019 Arab-wide meta-analysis estimated a pooled BRCA mutation rate of 22% in Gulf populations [22].

Incidence trends show Qatar and Kuwait now rival Bahrain, with age-standardized rates of 56.9 and 52.66 per 100,000, respectively [6], which is still below Bahrain’s 58.5 but above Saudi’s 19.8.

Collectively, our 39% P/LP prevalence exceeds regional averages, likely reflecting (i) stringent NCCN-based selection and (ii) adoption of broad 143-gene panels.

In general, the prevalence of a positive family history of any BC is around 15%. However, in this study, the majority (84%) of participants had a family history of BC, with 44% involving first-degree relatives. This high percentage can be attributed to the fact that the sample was preselected based on genetic testing criteria. Future research would benefit from comparing these values with a control group of women diagnosed with BC who do not meet the NCCN criteria.

Interestingly, family history was significantly higher (100%) in the disease-free group (*p* = 0.056). This aligns with expectations, since the participants were selected for genetic testing following the NCCN guidelines, which heavily emphasize family history. Most individuals in the disease-free group had a first-degree relative with breast, ovarian, pancreatic, or prostate cancer compared to only about one-third of those with cancer (*p* < 0.001) (Table 2). It is important to note that these participants were disease free at the time of the study. Prospective studies will be needed to determine whether they develop BC in the future.

Despite the importance of family history, some studies doubt relying solely on it. A prospective multicenter study, for instance, found that 48% of their patients have actionable mutations that would have been missed if they had followed only the phenotype or family history as the criterion for testing [23]. Therefore, genetic testing is encouraged regardless of family history.

Triple-negative BC (TNBC) is notorious for lacking receptors for estrogen, progesterone, and human epidermal growth factor receptor 2 (HER2), which makes it difficult to respond to conventional treatment. It accounts for 15% of all BCs [24]. In our study, only 11% of patients had TNBC. A few studies showed that having a first-degree family history of BC was associated with an increased risk of triple-negative BC [25]. In this study, the patients with moderate-risk mutations exhibited a higher incidence of TNBC compared to those with high-risk mutations—an unexpected and conflicting observation. This paradox may be attributed to the small sample size and potential referral bias.

The most common type of cancer in our sample was invasive ductal carcinoma, accounting for 74% of the cases, which is close to the international figure of 80% [26]. Invasive lobular carcinoma came in second (16%), and the international percentage is 10–15% [27]. Other less common types accounted for 9% of all tumors in our sample.

BRCA1 and BRCA2 mutations were broadly studied in terms of prevalence among patients with BC. Two remarkable observations among Ashkenazi Jews and Icelanders highlighted a high incidence of specific mutations in these genes. BRCA1 mutations were detected in 20% of patients diagnosed before the age of 42 years, and BRCA2 mutations were detected in 8% of the cases. In contrast, 24% of the women in Iceland with BC diagnosed before the age of 40 years had an isolated BRCA2 mutation [28,29]. Both populations represent a classic founder effect. In the UK, however, mutations of these genes were detected in around 6% of women diagnosed with BC before the age of 36 years (3.5% in BRCA1 and 2.4% in BRCA2), while in women diagnosed between the ages of 36 years and 45 years, 4% had mutations in BRCA1 and 2% had mutations in BRCA2 [30].

In this study, BRCA1 and BRCA2 mutations accounted for 31% of all mutations (17% in BRCA1 and 14% in BRCA2). This figure almost matches the 31.9% of mutations in BRCA1 and BRCA2 observed by an Indian study among 160 unrelated patients [31]. A recent study conducted in India on 155 patients found 83% of mutations were in these two genes (66% in BRCA1 and 17% in BRCA2) [32].

P/LP variants were detected in 39% of our sample, and 61% were VUS. A comparable result (41%) of P/LP was observed [32]. However, Chheda et al. reported a lower percentage for VUS (6.25%) [31]. This remarkable difference in results can be attributed to the extended nature of the NGS panel we used, as the higher the number of genes included, the more VUS can be expected. Furthermore, expansive NGS panels and under-representation of Arab genomes inflate the rate of VUS.

In our study, other pathogenic gene mutations (non-BRCA) accounted for 17%, which is comparable to other studies. For instance, studies reported 22.6%, 15%, and 11.9% [32,33,34], respectively. For other high-penetrance genes, only one (1%) mutation in the PT53 gene was detected. For moderate-penetrance genes, five (8%) mutations in the ATM gene were reported, and three (5%) mutations in the CHEK2 gene were reported.

In contrast to international figures, where high-penetrant genes contribute to around 25% of hereditary BC, in our study, they only accounted for a minority of 2%. Almost half of the variants detected were classified as low penetrance in this sample as compared to 70% internationally [35].

Regarding the pathogenicity of germline mutations and risk classification, we found that pathogenicity was significantly higher in individuals with high-risk mutations compared to those with medium- and low-risk mutations, supporting the accuracy of our genetic reports. However, mortality was highest among those with medium-risk mutations, exceeding that of both the high- and low-risk groups. This might be due to the limited sample size or delayed diagnosis and treatment.

## 5. Limitations

This study included all attendees of the genetics center who had their blood genetically tested during the study period, thereby minimizing selection bias. However, this study was limited to a specific timeframe, encompassing only attendees from two years. This restriction led to a smaller sample size, which might have impacted the precision and generalizability of our findings. Additionally, the retrospective nature of this study relied on existing records, which could introduce potential information bias due to incomplete or inaccurate documentation. Certain clinical data provided by the participants, such as family history and the specific types of cancers affecting family members, could not be independently verified, as some individuals received treatment outside the country, and corresponding medical records were unavailable.

Furthermore, the use of stringent criteria—such as the NCCN guidelines—by the genetic center to select eligible patients for genetic testing has limited the generalizability of our results to the broader local population. We also faced challenges, including incomplete treatment data, the absence of male cases, and limited regional genomic resources.

## 6. Recommendations

### 6.1. Public-Health Implications

Effective preventive programs at all levels—including awareness campaigns, screening, and rehabilitation—are essential. We strongly advocate for the widespread availability of genetic counseling and testing, given their critical role in prevention, diagnosis, and treatment. Bahrain’s young age at onset (<40 years) and the high frequency of pathogenic/likely pathogenic (P/LP) variants support the need for population-level risk assessment pathways, tiered genetic counseling models, and the integration of germline findings into multidisciplinary tumor boards across the GCC.

We also recommend incorporating risk-assessment tools into primary care, surgical, and oncology settings, with an emphasis on the importance of family history in breast and other hereditary cancers.

Regional collaborative efforts must be initiated, and pan-Arab variant curation networks must be established to help reduce the rate of variants of uncertain significance (VUS).

### 6.2. Future Research

We recommend conducting a larger, prospective study encompassing all patients referred for genetic testing, including male patients, with a focus on evaluating the effectiveness of cascade screening and genetic counseling. A comparative study should also be encouraged to compare the results with a matched control group of BC women without a family history of BC. As observed from this study, medium-risk mutations are significantly associated with a poorer prognosis. Therefore, further investigation is needed.

## 7. Conclusions

Genetic testing and counseling are increasingly recognized as vital components in BC management. In Bahrain, the genetic profile of BC germline mutations aligns closely with international data. This study is a milestone in understanding the genetic basis of breast cancer in Bahrain, offering actionable insights for early detection, not only for those with a family history but for all cancer patients. It also alerts the country to update the national cancer control strategies. Its findings are poised to improve testing policies.

This study lays the groundwork for further research into moderate- and low-risk mutations, particularly in ethnically diverse populations in Bahrain and the wider Middle East, where unique genetic variations may exist but are currently underexplored.

## Figures and Tables

**Table 1:** Characteristics of participants (n = 160).

Variable	Number or Mean (SD), Median	%
Status		
Affected with cancer	134	83.8
Asymptomatic (no cancer diagnosis) with strong family history	26	16.2
**Age (years), Mean (SD)**	54.58 (11.32), 56	
**Age categories (years)**		
20–29	4	2.5
30–39	9	5.6
40–49	42	26.3
50–59	50	31.3
≥60	55	34.4
**Age at diagnosis (years), Mean (SD)**	39.13 (19.15)	
**Age at diagnosis categories (years)**		
20–29	4	3
30–39	19	14
40–49	63	47
50–59	40	30
≥60	8	6
**Family history of Cancer**		
Yes	135	84
No	22	14
Missing	3	2
**Type of family history**		
No family history	22	14
One or more first degree with breast (age 50 years or less), ovarian, pancreatic, prostate, male breast	71	44
At least 3 with breast, prostate, ovarian, pancreatic on the same side of the family	8	5
1st or 2nd degree with colon or uterine CA	8	5
Any other blood relative with cancer	48	30
Missing	3	2
**Type of cancer in the participant**		
Breast	109	81
Ovarian	5	4
Both breast and ovarian	1	1
Breast and other combination	18	13
Ovarian and other combination	1	1
**Laterality and Recurrence**		
Bilateral and/or recurrent	30	23
Unilateral and/or no recurrent	101	77
**Triple negative**		
Yes	12	11
No	97	89
**Pathology**		
In situ intraductal	2	3
In situ lobular	1	1
Invasive ductal	54	74
Invasive lobular	12	16
Invasive medullary	0	0
Invasive papillary	1	1
Invasive tubular	0	0
Other	3	4
**Type of mutation**		
BRCA1	10	17
BRCA2	8	14
ATM	5	8
MSH2	3	5
CHEK2	3	5
PALB2	2	3
MSH6	1	2
TP53	1	2
Others	26	44
**Pathogenicity**		
Pathogenic	23	39
VUS	36	61
**Mutation Category**		
High risk	19	32
Moderate risk	10	17
Low risk	30	51
**Metastasis**		
Yes	22	16
No	111	83
No data available	1	1
**Status**		
Alive	151	94
Deceased	9	6

**Table 2:** Sociodemographic and clinical data by status of patient (affected vs. at-risk, cancer-free).

Variable	Status		*p*-Value
	Affected by Breast Cancer (n = 134) Mean (SD) or N (%)	No Cancer Diagnosis (n = 26) Mean (SD) or N (%)	
**Age (years), Mean (SD)**	56.63 (10.39)	43.48 (9.67)	0.509
**Age categories (years)**			
20–29	2 (1.5%)	2 (7.7%)	˂0.001
30–39	4 (3.0%)	5 (19.2%)	
40–49	29 (21.6%)	13 (50.0%)	
50–59	45 (33.6%)	5 (19.2%)	
≥60	54 (40.3%)	1 (3.8%)	
**Age at diagnosis (years), Mean (SD)**	46.37 (9.84)	NA	
**Age at diagnosis categories (years)**			
20–29	4 (3.0%)	NA	NA
30–39	19 (14.2%)		
40–49	63 (47.0%)		
50–59	40 (29.9%)		
≥60	8 (6.0%)		
**Family history of Cancer**			
Yes	109 (81.3%)	26 (100%)	0.056
No	22 (17.2%)	0	
Missing	3 (1.5%)	0	
**Type of family history**			
No family history	22 (16.4%)	0	˂0.001
One or more first degree with breast (age 50 years or less), ovarian, pancreatic, prostate, male breast	48 (35.8%)	23 (88.5%)	
At least 3 with breast, prostate, ovarian, pancreatic on the same side of the family	6 (4.5%)	2 (7.7%)	
1st or 2nd degree with colon or uterine CA	8 (6.0%)	0	
Any other blood relative with cancer	47 (35.1%)	1 (3.8%)	
Missing	3 (3.0%)	0	
**Type of cancer in the participant**			
Breast	109 (81.3%)	NA	NA
Ovarian	5 (3.7%)		
Both breast and ovarian	1 (0.7%)		
Breast and other combination	18 (13.4%)		
Ovarian and other combination	1 (0.7%)		
**Laterality**			
Yes	2 (1.5%)	NA	NA
No	30 (22.4%)		
Missing	102 (76.1%)		
**Triple negative**			
Yes	12 (9.0%)	NA	NA
No	97 (72.4%)		
Missing	20 (14.9%)		
Not applicable (Ovarian)	5 (3.7%)		
**Pathology**			
In situ intraductal	2 (1%)	NA	NA
In situ lobular	1 (1%)		
Invasive ductal	54 (40%)		
Invasive lobular	12 (9%)		
Invasive medullary	0		
Invasive papillary	1 (1%)		
Invasive tubular	0 (0%)		
Other	3 (2%)		
Missing	56 (42%)		
Not applicable (ovarian) *	5 (%)		
**Type of mutation**			
None	82 (61%)	19 (73%)	0.615
BRCA1	7 (5%)	3 (12%)	
BRCA2	7 (5%)	1 (4%)	
PALB2	2 (1%)	0 (0%)	
MSH2	2 (1%)	1 (4%)	
MSH6	1 (1%)	0 (0%)	
TP53	1(1%)	0 (0%)	
ATM	5 (4%)	0 (0%)	
CHEK2	3 (2%)	0 (0%)	
Others **	24 (18%)	2 (8%)	
**Metastasis**			
Yes	22 (16.4%)	NA	NA
No	110 (82.1%)		
Missing	2 (1.5%)		
**Status**			
Alive	125 (93.3%)	26 (100%)	0.194 ***
Dead	9 (6.7%)	0	
**Pathogenicity**			
Pathogenic	19 (14.2%)	4 (15.4%)	0.338
VUS	33 (24.6%)	3 (11.5%)	
No mutation	82 (61.2%)	19 (73.1%)	
Pathogenic Mutation Category **			
High risk	14 (10.4%)	4 (15.4%)	0.296
Moderate risk	10 (7.5%)	0	
Low risk	27 (20.1%)	3 (11.5%)	

* Participant had ovarian cancer. ** high risk is BRCA1 and 2; medium risk is ATM, CHEK2, PALB2, TP53. *** Fisher’s test.

**Table 3:** Sociodemographic and clinical data by status of patient (affected vs. at-risk, cancer-free).

Variable	Mutation Category				*p*-Value
	None	High Risk	Medium Risk	Low Risk	
Status					
Affected with cancer	83 (61.9%)	14 (10.4%)	10 (7.5%)	27 (20.1%)	0.296
At-risk, cancer-free (or benign) with strong family history	19 (73.1%)	4 (15.4%)	0	3 (11.5%)	
**Family history of Ca**					
Yes	83 (81.4%)	18 (100%)	10 (100%)	24 (80.0%)	0.304
No	17 (16.7%)	0	0	6 (20.0%)	
Not available	2 (2.0%)	0	0	0	
**Pathogenicity**					
Pathogenic	1 (1.0%)	13 (72.2%)	3 (30%)	6 (20.0%)	˂0.001
VUS	0	5 (27.8%)	7 (70%)	24 (80.0%)	
Not applicable	101 (99.0%)	0	0	0	
**Alive or dead**					
Alive	101(99.0%)	15 (83.3%)	8 (80%)	27 (90.0%)	0.004
Dead	1 (1.0%)	3 (16.7)	2 (20%)	3 (10.0%)	
**Type of family history**					
No history	16 (15.7%)	0	0	6 (20.0%)	0.033
One or more first degree with breast (age 50 years or less), ovarian, pancreatic, prostate, male breast	41 (40.2%)	11 (61.1%)	7 (70.0%)	12 (40.0%)	
At least 3 with breast, prostate, ovarian, panceatic on the same side of the family	5 (4.9%)	2 (11.1%)	0	1 (3.3%)	
1st or 2nd degree with colon or uterine CA	1 (1.0%)	1 (5.6%)	1 (10.0%)	5 (16.7%)	
Any other blood relative with cancer	36 (35.3%)	4 (22.2%)	2 (20.0%)	6 (20.0%)	
Not available	3 (2.9%)	0	0	0	
**Laterality**					
Yes	18 (17.6%)	5 (27.8%)	2 (20.0%)	5 (16.7%)	0.650
No	62 (60.8%)	9 (50.0%)	8 (80.0%)	22 (73.3%)	
Missing	2 (2.0%)	0	0	0	
Not applicable	20 (19.6%)	4 (22.2%)	0	3 (10.0%)	
**Triple negative**					
Yes	9 (8.8%)	1 (5.6%)	1 (10.0%)	1 (3.3%)	0.091
No	64 (62.7%)	7 (38.9%)	6 (60.0%)	20 (66.7%)	
Missing	7 (6.9%)	5 (27.8%)	3 (30.0%)	5 (16.7%)	
Not applicable	22 (21.6%)	5 (27.8%)	0	4 (13.3%)	
**Pathology**					
In situ intraductal	2 (2.0%)	0	0	0	0.876
In situ lobular	1 (1.0%)	0	0	0	
Invasive ductal	36 (35.3%)	4 (22.2%)	2 (20.0%)	12 (40%)	
Invasive lobular	7 (6.9%)	1 (5.6%)	2 (20.0%)	2 (6.7%)	
Invasive papillary	1 (1.0%)	0	0	0	
Other	2 (2.0%)	0	0	1 (3.3%)	
Missing	30 (22.5%)	8 (44.4%)	6 (60.0%)	11 (36.7%)	
Not applicable *	23 (29.4%)	5 (27.8%)	0	4 (13.3%)	
**Metastases**					
Yes	5 (4.9%)	4 (22.2%)	4 (40.0%)	9 (30.0%)	0.006
No	77 (75.5%)	10 (55.6%)	6 (60.0%)	18 (60.0%)	
Missing	2 (2.0%)	0	0	0	
Not applicable	18 (17.6%)	4 (22.2%)	0	3 (10.0%)	

* Participants with no history of cancer.

**Table 4:** Association between mutation category and age variables using ANOVA.

Variable	Mutation Category	N	Mean (SD)	95% CI	*p*-Value
Age at diagnosis	No mutation	102	37.38 (19.68)	(33.52–41.25)	0.250
	High risk	18	37.44 (22.39)	(26.31–48.58)	
	Medium risk	10	46.60 (5.19)	(42.89–50.31)	
	Low risk	30	43.57 (17.56)	(37.01–50.13)	

## Data Availability

The data presented in this study are available on request from the corresponding author upon approval from the Government Hospitals Administration.

## Abbreviations

ASR	age-standardized incidence rate
BC	breast cancer
GCC	Gulf Cooperation Council
GH	Governmental Hospitals
HBOC	hereditary breast and ovarian cancer
HER2	human epidermal growth factor receptor 2
NCCN	National Comprehensive Cancer Network
NGS	next generation sequencing
P/LP	pathogenic and likely pathogenic
TNBC	triple-negative breast cancer
VUS	variant of unknown significance
WHO	World Health Organization

## References

[1] Sedeta E.T., Jobre B., Avezbakiyev B. (2023). Breast cancer: Global patterns of incidence, mortality, and trends. J. Clin. Oncol..

[2] Rudolph A., Chang-Claude J., Schmidt M.K. (2016). Gene–environment interaction and risk of breast cancer. Br. J. Cancer.

[3] Yoshida R. (2021). Hereditary breast and ovarian cancer (HBOC): Review of its molecular characteristics, screening, treatment, and prognosis. Breast Cancer.

[4] Ferlay J., Ervik M., Lam F., Laversanne M., Colombet M., Mery L., Piñeros M., Znaor A., Soerjomataram I., Bray F. (2024). Global Cancer Observatory: Cancer Today (Version 1.1).

[5] Hamadeh R.R., Abulfatih N.M., Fekri M.A., Al-Mehza H.E. (2014). Epidemiology of Breast Cancer among Bahraini Women: Data from the Bahrain Cancer Registry. Sultan. Qaboos Univ. Med. J..

[6] Chaabna K., Ladumor H., Cheema S. (2023). Ecological study of breast cancer incidence among nationals and nonnationals in the Gulf Cooperation Council countries. East. Mediterr. Health J..

[7] Gabet S., Lemarchand C., Guénel P., Slama R. (2021). Breast Cancer Risk in Association with Atmospheric Pollution Exposure: A Meta-Analysis of Effect Estimates Followed by a Health Impact Assessment. Environ. Health Perspect..

[8] Albeshan S.M., Mackey M.G., Hossain S.Z., Alfuraih A.A., Brennan P.C. (2018). Breast cancer Epidemiology in Gulf Cooperation Council Countries: A regional and international comparison. Clin. Breast Cancer.

[9] AlZaman A.S., Mughal S.A., AlZaman Y.S., AlZaman E.S. (2016). Correlation between hormone receptor status and age, and its prognostic implications in breast cancer patients in Bahrain. Saudi Med. J..

[10] Liu T., Yu J., Gao Y., Ma X., Jiang S., Gu Y., Ming W.-K. (2024). Prophylactic Interventions for Hereditary Breast and Ovarian Cancer Risks and Mortality in BRCA1/2 Carriers. Cancers.

[11] Nielsen F.C., van Overeem Hansen T., Sørensen C.S. (2016). Hereditary breast and ovarian cancer: New genes in confined pathways. Nat. Rev. Cancer.

[12] Guindalini R.S.C., Viana D.V., Kitajima J.P.F.W., Rocha V.M., López R.V.M., Zheng Y., Freitas É., Monteiro F.P.M., Valim A., Schlesinger D. (2022). Detection of germline variants in Brazilian breast cancer patients using multigene panel testing. Sci. Rep..

[13] Daly M.B., Pal T., Berry M.P., Buys S.S., Dickson P., Domchek S.M., Elkhanany A., Friedman S., Goggins M., Hutton M.L. (2021). Genetic/Familial High-Risk Assessment: Breast, Ovarian, and Pancreatic, Version 2.2021, NCCN Clinical Practice Guidelines in Oncology. J. Natl. Compr. Cancer Netw..

[14] Pintican R.M., Chiorean A., Duma M., Feier D., Szep M., Eniu D., Goidescu I., Dudea S. (2022). Are Mutation Carrier Patients Different from Non-Carrier Patients? Genetic, Pathology, and US Features of Patients with Breast Cancer. Cancers.

[15] Al Hannan F., Keogh M.B., Taha S., Al Buainain L. (2019). Characterization of *BRCA1* and *BRCA2* genetic variants in a cohort of Bahraini breast cancer patients using next-generation sequencing. Mol. Genet. Genom. Med..

[16] Al-Kafaji G., Jassim G., AlHajeri A., Alawadhi A.M.T., Fida M., Sahin I., Alali F., Fadel E., Reed A.M. (2023). Investigation of germline variants in Bahraini women with breast cancer using next-generation sequencing based-multigene panel. PLoS ONE.

[17] Walsh T., Lee M.K., Casadei S., Thornton A.M., Stray S.M., Pennil C., Nord A.S., Mandell J.B., Swisher E.M., King M.-C. (2010). Detection of inherited mutations for breast and ovarian cancer using genomic capture and massively parallel sequencing. Proc. Natl. Acad. Sci. USA.

[18] de Jong M.M., Nolte I.M., Te Meerman G.J., van der Graaf W.T., Oosterwijk J.C., Kleibeuker J.H., De Vries E.G.E. (2002). Genes other than BRCA1 and BRCA2 involved in breast cancer susceptibility. J. Med. Genet..

[19] WHO (2019). Breast Tumours, WHO Classification of Tumours, 2019.

[20] Ekram S.N., Elemam O., Alandonisi M., Flemban A., Samkari J., Zainuddin H.H., Azher Z., Tashkandi E., Mufti A., Khogeer A. (2025). Mutational spectrum and profile of breast and ovarian cancer patients in Saudi Arabia’s western region: Single center experience. Discov. Oncol..

[21] Al Ali A.A.A.A., Al Ali M.M.A., El-Shourbagy D.M.A., Tirmazy S.H.H., Mirza I., Raza A., Latif M.F., Yasaei H. (2025). Genetic character-ization of BRCA1 and BRCA2 variants in cancer and high-risk family screening cohorts in the UAE population. J Cancer Res. Clin. Oncol..

[22] Abdulrashid K., AlHussaini N., Ahmed W., Thalib L. (2019). Prevalence of BRCA mutations among hereditary breast and/or ovarian cancer patients in Arab countries: Systematic review and meta-analysis. BMC Cancer.

[23] Samadder N.J., Riegert-Johnson D., Boardman L., Rhodes D., Wick M., Okuno S., Kunze K.L., Golafshar M., Uson P.L.S., Mountjoy L. (2021). Comparison of Universal Genetic Testing vs Guideline-Directed Targeted Testing for Patients With Hereditary Cancer Syndrome. JAMA Oncol..

[24] de Paula B., Crocamo S., de Sousa C.A.M., Valverde P., Rezende F., Abdelhay E. (2024). Triple-Negative Breast Cancer Subclassified by Immunohistochemistry: Correlation with Clinical and Pathological Outcomes in Patients Receiving Neoadjuvant Chemotherapy. Int. J. Mol. Sci..

[25] Phipps A.I., Buist D.S.M., Malone K.E., Barlow W.E., Porter P.L., Kerlikowske K., Li C.I. (2011). Family history of breast cancer in first-degree relatives and triple-negative breast cancer risk. Breast Cancer Res. Treat..

[26] Elston C.W., Ellis I.O. (1991). Pathological prognostic factors in breast cancer. I. The value of histological grade in breast cancer: Experience from a large study with long-term follow-up. Histopathology.

[27] Li C.I., Anderson B.O., Daling J.R., Moe R.E. (2003). Trends in incidence rates of invasive lobular and ductal breast Carcinoma. JAMA.

[28] Neuhausen S., Gilewski T., Norton L., Tran T., McGuire P., Swensen J., Hampel H., Borgen P., Brown K., Skolnick M. (1996). Recurrent BRCA2 6174delT mutations in Ashkenazi Jewish women affected by breast cancer. Nat. Genet..

[29] Thorlacius S., Sigurdsson S., Bjarnadottir H., Olafsdottir G., Jonasson J.G., Tryggvadottir L., Tulinius H., E Eyfjörd J. (1997). Study of a single BRCA2 mutation with high carrier frequency in a small population. Am. J. Hum. Genet..

[30] Peto J., Collins N., Barfoot R., Seal S., Warren W., Rahman N., Easton D.F., Evans C., Deacon J., Stratton M.R. (1999). Prevalence of BRCA1 and BRCA2 Gene Mutations in Patients With Early-Onset Breast Cancer. JNCI J. Natl. Cancer Inst..

[31] Chadha K., Chheda P., Pande S., Dama T., Vinarkar S., Chanekar M., Limaye S., Shah N. (2020). Spectrum of germline BRCA mutations in hereditary breast and ovarian cancer syndrome in Indian population: A central reference laboratory experience. Cancer Res. Stat. Treat..

[32] Pramanik R., Chitikela S., Deo S., Gogia A., Batra A., Kumar A., Gupta R., Thakral D., Ramprasad V.L., Mathur S. (2024). Comprehensive germline profiling of patients with breast cancer: Initial experience from a Familial Cancer Clinic. Ecancermedicalscience.

[33] Singh J., Thota N., Singh S., Padhi S., Mohan P., Deshwal S., Sur S., Ghosh M., Agarwal A., Sarin R. (2018). Screening of over 1000 Indian patients with breast and/or ovarian cancer with a multi-gene panel: Prevalence of BRCA1/2 and non-BRCA mutations. Breast Cancer Res. Treat..

[34] Kadri M.S.N., Patel K.M., Bhargava P.A., Shah F.D., Badgujar N.V., Tarapara B.V., Patel P.S., Shaikh M.I., Shah K., Patel A. (2021). Mutational Landscape for Indian Hereditary Breast and Ovarian Cancer Cohort Suggests Need for Identifying Population Specific Genes and Biomarkers for Screening. Front. Oncol..

[35] Rizzolo P., Silvestri V., Falchetti M., Ottini L. (2011). Inherited and acquired alterations in development of breast cancer. Appl. Clin. Genet..

